# Implementation challenges for an ethical introduction of noninvasive prenatal testing: a qualitative study of healthcare professionals’ views from Lebanon and Quebec

**DOI:** 10.1186/s12910-020-0455-x

**Published:** 2020-02-10

**Authors:** Hazar Haidar, Meredith Vanstone, Anne-Marie Laberge, Gilles Bibeau, Labib Ghulmiyyah, Vardit Ravitsky

**Affiliations:** 1 Institute for Health and Social Policy, McGill University, Montreal, Canada; 2 Department of Family Medicine, McMaster Program for Education Research, Innovation and Theory, McMaster University, Hamilton, Canada; 3 Medical Genetics, Department of Pediatrics, and Research Center, Centre Hospitalier Universitaire Sainte-Justine, Montreal, Canada; 4 Department of Pediatrics, Faculty of Medicine; and Department of Social and Preventive Medicine, École de Santé Publique, Université de Montréal, Montreal, Canada; 5 Department of Anthropology, Faculty of Arts and Sciences, Université de Montréal, Montreal, Canada; 6 Department of Obstetrics and Gynecology, American University of Beirut, Beirut, Lebanon; 7 Bioethics Program, Department of Social and Preventive Medicine, School of Public Health, Université de Montréal, Montreal, Canada

**Keywords:** Non-invasive prenatal testing (NIPT), Implementation, Lebanon, Quebec, Qualitative interview study, Ethical introduction

## Abstract

**Background:**

The clinical introduction of non-invasive prenatal testing for fetal aneuploidies is currently transforming the landscape of prenatal screening in many countries. Since it is noninvasive, safe and allows the early detection of abnormalities, NIPT expanded rapidly and the test is currently commercially available in most of the world. As NIPT is being introduced globally, its clinical implementation should consider various challenges, including the role of the surrounding social and cultural contexts. We conducted a qualitative study with healthcare professionals in Lebanon and Quebec as case studies, to highlight the relevance of cultural contexts and to explore the concerns that should be taken into account for an ethical implementation of NIPT.

**Methods:**

We conducted semi-structured interviews with 20 healthcare professionals (HCPs), 10 from each country, practicing in the field of prenatal screening and follow up diagnostic testing, including obstetricians and gynecologists, nurses, medical geneticists and, genetic counselors. We aimed to 1) explore HCPs’ perceptions and views regarding issues raised by NIPT and 2) to shed light on ways in which the introduction of the same technology (NIPT) in two different contexts (Lebanon and Quebec) raises common and different challenges that are influenced by the cultural norms and legal policies in place.

**Results:**

We identified challenges to the ethical implementation of NIPT. Some are common to both contexts, including financial/economic, social, and organizational/ educational challenges. Others are specific to each context. For example, challenges for Lebanon include abortion policy and financial profit, and in Quebec challenges include lobbying by Disability rights associations and geographical access to NIPT.

**Conclusions:**

Our findings highlight the need to consider specific issues related to various cultural contexts when developing frameworks that can guide an ethically sound implementation of NIPT. Further, they show that healthcare professional education and training remain paramount in order to provide NIPT counseling in a way that supports pregnant women and couples’ choice.

## Background

### Cross-cultural perspectives on the ethical implementation of NIPT in Quebec and Lebanon

This article explores and compares the views of healthcare professionals from Lebanon and Quebec regarding the challenges raised by the clinical implementation of a fast-moving technology: non-invasive prenatal testing (NIPT). NIPT involves the analysis of cell-free DNA fragments present in the maternal plasma [1] to detect the likelihood of fetal genetic aneuploidies such as trisomy 21, 13 and 18, and sex chromosome anomalies [2]. NIPT can also be used to screen for non-medical genetic information such as fetal sex. NIPT can be performed as early as 9 weeks’ gestation [3, 4] and is considered safe because it requires only a blood draw that can be obtained without the risk of miscarriage associated with invasive procedures (such as amniocentesis and chorionic villus sampling (CVS)) [5]. NIPT is a screening test and not a diagnostic test, meaning that it does not provide a definitive answer about whether or not a fetus has a genetic anomaly. Irrevocable decisions about the pregnancy should only be made after confirmation by a diagnostic test. Compared to existing screening tests, NIPT is considered superior in terms of accuracy because it can detect some conditions with high sensitivity (true positive rate) and specificity (true negative rate) [6]. For instance, it enables the screening of Down syndrome with 99.21% sensitivity and 99.95% specificity.

Unlike other prenatal tests, NIPT’s development and clinical introduction has been driven by industry and market pressures from various companies wishing to capture a market share. Consequently, NIPT is often described in industry-produced materials as having very favourable sensitivity and specificity rates, rather than having potentially more useful indicators such as positive predictive value [7, 8]. The rapid implementation of NIPT in over 60 countries [9] has been facilitated by “aggressive advertising” [7] p.849. The rapid pace, as well as the context in which NIPT is emerging, raise important ethical, legal and policy issues. For instance, the high cost of NIPT limits access for those without financial resources, creating inequity [10, 11]. Since it became commercially available in 2011, the price of NIPT has quickly declined [12], but NIPT cost is a key challenge, amongst others, to an ethical implementation of NIPT in different countries.

The literature addressing these challenges has consisted mainly of literature reviews [9, 13], quantitative studies in various countries [14–17], or qualitative studies performed in a specific country e.g. The Netherlands [18]. A comparative cultural perspective is largely missing and can be helpful in shedding light on practical concerns and context-specific challenges raised by NIPT. In previous work, we highlighted cross-cultural differences in women and couples’ decision-making about NIPT, suggesting that differences in implementation have important ethical implications [19]. We showed, for instance, how religious beliefs held by pregnant women and partners in Lebanon played a significant role in accepting or declining NIPT.

This paper focuses on the views of healthcare professionals (HCPs) from Lebanon and Quebec, highlighting the impact of cultural factors and norms on the attitudes and perceptions of HCPs towards challenges raised by and barriers to an ethical implementation of NIPT. This comparison is timely as NIPT is changing the landscape of prenatal screening by offering clinical benefits compared with traditional prenatal tests. The aim of this comparison is not to point out what specific elements (such as the potential increase in pregnancy terminations) are considered by HCPs to be ethical or unethical, but rather to understand how cultural values, norms and resulting policies (Table 1) shape the perceived differences and similarities of the challenges raised by NIPT’s clinical implementation. We conducted 20 semi-structured interviews with HCPs (10 in each country), to explore their views regarding the challenges to the clinical implementation of NIPT and to shed light on the way in which local cultural and legal factors influence the introduction of the same technology in two different contexts.

**Table 1:** Summary of the contextual background for Quebec and Lebanon^a^

Country	Quebec	Lebanon
Structure of the healthcare system	Public; meaning that the State and therefore the provincial government being the principal administrator of healthcare services.	Private and public; meaning that the provision of healthcare services includes payers and providers from both the public and the private sectors with the latter being the main provider of healthcare services in the country [20].
Coverage of prenatal tests	Prenatal tests are covered by the healthcare system for pregnant women if medically indicated and prescribed by the physician. As for NIPT, it is covered in few Canadian provinces such as Ontario and British Colombia for women with high-risk pregnancies. However, in Quebec, the decision has been made to cover it for high-risk pregnancies but has not yet entered into effect.	Prenatal tests are generally not covered unless one benefits from a public or a private coverage. Regarding NIPT, it is not yet reimbursed by any form of coverage and is paid for privately.
The legal status of abortion	Abortion in Quebec is legal at any point during pregnancy with late term abortions e.i. after 24 weeks, performed in few clinical cases and in restricted number of institutions [21].	Abortion in Lebanon is illegal at any point in time except if the mother’s life is in danger. Nevertheless, the law is not enforced and it’s being clinically practiced in a clandestine manner in clinics or in hospital operation rooms [22].

^a^Readers interested in a more detailed description of the cultural contexts, health care settings and socio-cultural practices relevant to NIPT in Lebanon and Quebec, will find this information in a previous publication entitled: Cross-cultural perspectives on decision making regarding noninvasive prenatal testing: A comparative study of Lebanon and Quebec. AJOB empirical bioethics. 2018 Apr-Jun;9(2):99–111

## Methodology

### Study design

We used a qualitative description (QD) methodology [23] that has been described in detail elsewhere [19]. Briefly, QD provides rich information regarding issues that are grounded in environmental and cultural contexts [24] and allows to answer questions of relevance to practitioners and policy makers [23]. We used QD to explore cultural nuances pertaining to implementation challenges for an ethical introduction of NIPT in two different contexts, Lebanon and Quebec, through a rich description emerging from the voices of HCPs. Based on HCPs’ views, the descriptions of the implementation pressures for NIPT clinical introduction will therefore be framed by the surrounding cultural contexts. These results might be used to inform practitioners as well as policy decision-makers about challenges raised by NIPT clinical implementation, thus supporting the usefulness of QD in our study.

### Sampling and recruitment

HCPs in the field of prenatal screening and follow-up diagnostic testing from Centre hospitalier universitaire Sainte-Justine (CHUSJ), Centre hospitalier de l’Université de montréal (CHUM) and American University of Beirut Medical Center (AUBMC) were invited to participate in our study. These HCPs were identified through collaborators who provided a list of HCPs eligible to participate. We also searched online for HCPs practicing in the field of prenatal testing in the above-mentioned institutions. HCPs were then invited by email to participate. In total, 40 HCPs were invited: 25 Quebecois and 15 Lebanese. We interviewed all those who agreed to participate. 10 Quebecois HCPs were interviewed between October 2014 and February 2015, and 10 Lebanese HCPs were interviewed between May 2015 and August 2015 (Table 2). We stopped recruitment after we reached data saturation.

**Table 2:** Participant Demographic Details

Total participants	Quebec	Lebanon
Data collection		
Interview participants	10	10
Profession		
Registered nurse	2	3
Medical geneticist	4	0
Obstetrician/Gynecologist	3	7
Genetic counselor	1	–
Gender		
Female	8	4
Male	2	6

### Data collection

In Montreal, one researcher (H.H.) conducted the semi-structured interviews with two registered nurses, four medical geneticists, three obstetricians/gynaecologists and one genetic counselor recruited from two different departments: the medical genetics division in the department of pediatrics and the department of obstetrics and gynecology. The interviews were conducted in French and face-to-face at the respondents’ workplaces.

In Beirut, the same researcher (H.H.) conducted the semi-structured interviews with three registered nurses and seven obstetricians/gynaecologists. All interviews were conducted in English, which was the preference expressed by HCPs and face to face at the respondents’ workplaces. We should note that in Lebanon there is a complete absence of genetic counseling services (Nakouzi, 2015) and therefore of genetic counselors.

We used the same interview guides in Montreal and Beirut that explored the same topics, such as general attitudes regarding NIPT and NIPT coverage. We modified a few questions in order to fit the appropriate context. For instance, we reformulated the question about the coverage of NIPT to refer to the healthcare system in place for each setting (public vs. private healthcare system). The interview guide is provided as a supplementary file to this manuscript (see Additional file 1). Interviews were audiotaped, transcribed verbatim, and anonymized. The symbols we used to anonymize our data refer to the following: HP: Health professional, Qc: Quebecois, and Lb: Lebanese.

### Data analysis

We used thematic analysis to perform our data analysis, facilitated by NVivo 11 the software package. Two independent researchers (H.H and G.B.) coded the interviews in Montreal and in Lebanon, compared the coded transcripts, and discussed the differences until they reached a consensus. H.H. translated the themes and selected quotes from the French interviews into English, to discuss the analysis content and to draft the manuscript. G.B. validated all the translations.

## Results

### Key findings

HCPs were asked to reflect about the ethical implementation challenges and barriers that should be taken into account for an ethical implementation of NIPT into the clinical encounter. The findings revealed remarkably similar themes within all professional groups, despite diverse backgrounds (medical geneticists, obstetricians and gynecologists, nurses and genetic counselors). Therefore, the findings are presented by theme, rather than by professional group. We distinguished two sets of ethical implementation challenges A) those that we found to be common to both contexts and that we grouped under the theme “common ethical implementation challenges” including three subthemes: financial/economic, social, and organizational/educational challenges; and B) those that are specific to each of the contexts and that we grouped under the theme “specific ethical implementation challenges” including abortion policy and HCPs’ financial gain in Lebanon, and the lobbying of disability rights’ associations and the geographical access challenges for Quebec.

### Ethical implementation challenges

#### Common ethical implementation challenges

##### Financial/economic challenges

The first and most often mentioned implementation concern by HCPs in both countries was the current high cost of NIPT. At the time of data collection, NIPT cost CAD 800 (or USD600 at the time) in Montreal and USD800 in Lebanon, and it was paid for privately in both settings.

Quebec has a public healthcare system and patients and providers are accustomed to having medically indicated tests (including other forms of prenatal screening) covered by public health insurance. Given this context, HCPs approached the issue of NIPT cost from the angles of equal access and justice. They raised concerns about the high cost of NIPT and the way it might create inequalities and discrimination between the people who would like to choose NIPT, since only those who can afford it have access.

*[ … ] the concern of inequality, because the test costs $ 800.00 and there are many people who cannot afford it, who would like to choose that option [NIPT testing], but who cannot afford it because of the cost. So, given their financial situation, they must put their pregnancy at risk by doing an invasive test. So that's the most difficult thing we face when meeting patients. (HP 5, Qc)*

Other HCPs were concerned about implications of cost for the Quebec public healthcare system and argued that the allocation of healthcare resources should be considered if NIPT is to be offered to all pregnant women.

*There is the economic barrier. Basically, this is a test that is currently expensive, so the healthcare system probably, for the moment, does not have the means; if we say that there are 80,000 births per year in Quebec, to offer the test to 80,000 women who will give birth, per year. So, I think it's mostly the economic aspect that constitutes a barrier. (HP 9, Qc)*

In Lebanon, HCPs cited the cost of NIPT as an important limitation for its ethical implementation. They argued that the introduction of NIPT will exacerbate the existing lack of coverage for prenatal screening tests in place. Further, they voiced concerns about private insurance companies who might not be willing to cover such an expensive test or who will only cover it for women with high-risk pregnancies:

*I mean in Lebanon, there is couple of things. There is first, let’s say the monetary problem so the money covering the test, I mean there is nothing really covered by insurance, so how about a test that is five times more expensive than regular screening tests is gonna be covered by insurance. That’s like a huge limitation. (HP 15, Lb)*

*Cost, only cost. It all amounts to money. Especially with government’s decisions and with marketing. You cannot now come with this cost, come to the government or to private insurance companies and tell them you should cover the NIPT instead of MSS test. There is a huge difference in price. (HP14, Lb)*

##### Societal challenges

HCPs in both Lebanon and Quebec raised concerns about the routine offer of NIPT and the negative consequences it might have on pregnant women and on society at large. For instance, in Quebec, a HCP mentioned the negative influence of NIPT becoming a routine test on pregnant women’s choice to consider the test and the “pressure” they might face to do the test:

*... It's also a bit like the screening we have right now, the doctors are offering it routinely and the patients who refuse the test are a little marginalized. It is written everywhere in the doctors' notes: "screening refused by the patient", underlined three times. So, for the doctors, instinctively, it is something that should be done by the patients. So sometimes I think that there is still the pressure to have to do this test ... So I think that it is the kind of issues that we could see, the fact that the doctors push you really for the test (HP 5, Qc)*

Another cited barrier resulting from NIPT becoming a routine test is the increase in pregnancy terminations and the decrease in the number of people with disability, which might therefore result in less tolerance for these people and invigorate the discrimination and stigmatization that they already face. Although this issue was raised by HCPs in both countries, it was much less of a concern for HCPs in Lebanon, who stated that the religious beliefs held by patients play a role in declining the termination of affected pregnancies.

*… it will make it easier to detect more of the chromosomal abnormalities because with the other tests yes we are missing some stuff … if it will become a routine and you will be catching all those, it might have an impact. But in our society, that impact would be a bit limited compared to the west because we still know of patients who have known that this is a trisomic baby and they elected not to terminate based on their religious beliefs. So it might just be preparing them for a chromosomal abnormal baby rather than letting them make decisions to terminate that pregnancy. (HP 16 , Lb)*

However, some Lebanese HCPs discussed concerns of pregnancy terminations in the context of “*misusing the test”* for sex selection and therefore “*terminations for the wrong gender” (HP 13, Lb)*:

*So my concern with this test, if it is widely available is really sex selection, so if we start to use NIPT at 9 weeks to determine sex selection and we, you know, society may suffer if we end up doing terminations for the wrong gender. So this is a misuse of the procedure or of the test … I think in any society, but definitely in societies where, you know, there’s male favoritism, let’s put it that way, such societies or cultures exactly are more, there might be, might be potential use of this test for that reason. I think it depends on exactly the geographic location in Lebanon but I can see it honestly both ways, it could be used for someone who’s had 3 girls and they’re looking for the boy or vice versa, for someone 3 boys and looking for the girl (HP 13, Lb)*

Nevertheless, HCPs in Quebec went on to say that if NIPT becomes a routine test, it will lead to eugenics because “*as a society we will eliminate certain genetic particularities* and *that’s where we’re going, the perfect child” (HP 7, Qc)*:

*We make the selection, so euh ... we call it eugenics. That's what we’re doing now with trisomy 21, with the screening program we’re using, so we continue in the same wave. We decide as a society that we will eliminate certain genetic particularities, including trisomy21, so ... If we talk about cystic fibrosis, if we talk about Duchenne disease, all diseases, and the more we advance, the more we have genetic markers, so where is the end of this screening? Of this selection? I do not know it, but that's where we’re going, the perfect child. (HP 7, Qc)*

It is feared that if NIPT becomes widely available, it will be considered as “just another blood test” [25] and hence the pregnant woman might perform it without fully considering its implications. This problem from the literature was brought up by a HCP in Quebec who expressed concerns about trivializing the offer of NIPT by thinking of it as another routine blood test in pregnancy:

*So the limits that are expected when this test becomes more available to the general population, I'm afraid that it would be like a test on a form paper, like we’re proposing a CBC (complete blood count), we check on the form NIPT, and the pregnant woman is going to do the NIPT, then the result comes out and it is trivialized without information, without signed consent or explanations (HP 1, Qc)*

##### Organizational and educational challenges

Training and educating HCPs to offer NIPT in a way that supports pregnant women and couples’ informed choice is of crucial importance for its ethical implementation. However, HCPs in Lebanon and Quebec showed concerns regarding HCPs lack of knowledge about the scientific properties of the test such as its sensitivity, specificity, and the fact it is a screening rather than a diagnostic test, and therefore the consequences of not providing appropriate counseling to patients. They emphasized the need of HCPs to be very well informed about the test, the procedure, its advantages and disadvantages, in order to communicate the information in a clear and concise manner.

*So non-optimal counseling, so if our health care providers are not well equipped to counsel patients appropriately this is a main barrier, the second barrier is cost, those are the two main ones. So, this test is very new and you know, not every provider is aware of the power of this test, its positives, its negatives, what’s the specificity, the sensitivity, the down side to, is it a screening test, is it a confirmatory test, so by that same token if the physicians are not well equipped, this is one of the main barriers, or don’t even know about it, there are doctors who do not know about it, a big portion of doctors still don’t know about it, the test. (HP 13, Lb)*

*Perhaps the staff who offers the ..., who informs the patients, who themselves are not well informed. I think that, it could be a barrier, a consideration. Physicians or nurses, in some clinics it can be nurses who make the first appointment of pregnancy monitoring. If they themselves are not well informed, they will not be able to adequately inform the patients. It can be a barrier, I think. It would especially be that. (HP 4, Qc)*

In addition to educating HCPs, both in Lebanon and Quebec, professionals thought that the lack of qualified HCPs including among others, geneticists and genetic counselors, constitute an important obstruct to NIPT ethical implementation especially if the test is to be offered to all pregnant women in the future.

*And another implementation consideration, the limits in the resources that are available in terms of genetic counselors and medical geneticists. Obviously, we cannot offer genetic counseling for all the women who will do this test, which may be desirable, but which is currently impossible. So at least we have to make sure that for all the doctors who are going to offer this test that the information is well understood. (HP 9, Qc)*

#### Specific ethical implementation challenges

##### Lebanon

**Abortion policy.**

Abortion is inherent to decision-making surrounding prenatal testing since couples/ parents might decide to continue or terminate a pregnancy based on the results of the prenatal test in question. Abortion policy is thus a crucial factor when considering the clinical and ethical implementation of a prenatal test such as NIPT.

Under the Lebanese penal code, abortion is legally prohibited at any point during pregnancy, and is allowed solely to save the mother’s life [26]. However, in the clinical practice, abortions are being performed regularly and clandestinely [22]. Given this context, abortion policy constitutes a barrier to an ethical implementation of NIPT insofar as couples and pregnant women will have the choice to accept or decline NIPT, but without having the choice to terminate the pregnancy -if they decided to do so- in a legal and safe manner.

The Lebanese abortion policy was brought up as a barrier to NIPT ethical implementation by only two of ten participants. This could be explained by the fact that abortion is clandestinely performed in clinics and thus participants did not consider it to be a barrier. Another reason that might explain this finding is that HCPs were avoiding an explicit discussion of the clinical status of abortion for fear of being prosecuted by justice for infringing the law:

*… Third by law in Lebanon, there is no termination of pregnancy. [Interviewer: by law, but we know it’s done.] it’s done, but by law it’s not allowed. So, we cannot go say it’s clinically done because we will go to jail. Yes, so basically those are the limitations of these tests in Lebanon and we’re still gonna do it. One day the law will change but, but again the culture is not gonna change, and … there is a huge chunk of our population that want to do nothing about this, even if it’s abnormal. (HP 15, Lb)*

*I think right now the cost mostly is the main problem, I think another thing that we were recently discussing is the law in Lebanon about abortion, it needs to be modified, right now it is illegal to perform an elective abortion even if the baby has a lethal anomaly like trisomy 13, trisomy 18. So you start thinking about what options do you have to offer the patient if you do such a test and it tells you that the baby has a lethal trisomy and then you confirm it with amniocentesis and then what? What do you do? Legally in Lebanon, up until now, you can’t offer an elective abortion for these women, so they would have to, if that’s what they want to do, they have to travel abroad to get an abortion and they can’t do it in Lebanon. So, this becomes another point of controversy (HP 20, Lb)*

**HCP financial gain related to NIPT uptake.**

Considering the Lebanese context and the significant influence of pharmaceutical companies’ marketing strategies on physicians’ behaviors to prescribe drugs, this situation might be extrapolated to the NIPT, which is being marketed directly to physicians. For instance, the American University of Beirut Medical Center has a contract with three companies: Centogene, Natera, and Illumina and the companies’ representatives usually meet with the physicians in order to market their tests.

Therefore, a Lebanese HCP feared that there might be a potential surge in NIPT prescriptions by HCPs seeking financial profit from the test:

*An important consideration, I fear that, because of the marketing and the companies who are in competition to sale their products, NIPT becomes a “business” through which the doctors prescribe more and more of the NIPT tests, so they make a financial profit. (HP 7, LB)*

##### Quebec

**Lobbying of disability rights associations in Quebec.**

The Trisomy 21 Prenatal Screening Program of Québec, implemented in 2012, created extremely strong opposition among many disability rights associations. They considered the implementation of such a program to be reinforcing the underlying eugenic presumptions inherent in prenatal testing, by contributing to the stigmatization of people with disabilities and their families and sending them a message that they are not welcome in society.

Some HCPs stated that if NIPT is implemented at the governmental level, it will generate the same negative social reactions as was the case with the Prenatal Screening Program, and that this will pose a barrier for its implementation:

*people will oppose it like has been done with screening ..., it was very long in Quebec before we went to the screening tests for Trisomy 21 because there was a lot of lobbying of trisomy 21 associations who stepped up to the barricades and said that we want to eliminate trisomy 21. There was a lot, a lot, a lot of resistance at that level. So if we’re going to offer things to target people with cystic fibrosis, for things like that or other, there will be a rise in these associations, which will make it more difficult to integrate, to have the test available at the governmental level (HP 7, Qc)*

**Geographical access to the test.**

Several HCPs in Quebec were concerned about the role that the geographical location of the pregnant woman would play in accessing NIPT. One HCP illustrated this concern by providing the example of nuchal translucency, where women who live outside the Montreal area might not be able to access it because of lack of accredited radiologists who can perform the technique:

*So, will all women outside Montreal be offered the NIPT? So, there should be universality of NIPT testing, no matter where the woman lives. It is the same problem with nuchal translucency, it is not all the women who have access to the nuchal translucency. In Montreal, it is much easier than if you are in Abitibi or in other areas where there may not be accredited radiologists to perform this technique. (HP 9, Qc)*

## Discussion

In this paper, we report implementation challenges for an ethical integration of NIPT into the clinic, as perceived by HCPs from Lebanon and Quebec. Although our participants were enthusiastic about the test and its clinical advantages, especially when they compared it to existing prenatal screening tests, they highlighted a variety of challenges and barriers to its clinical implementation. Many of these identified challenges are common to both contexts, such as financial, societal and educational concerns, while others are more specific to each setting.

Among the common ethical challenges, cost was cited as the crucial challenge for NIPT’s clinical implementation. This is consistent with other studies, emphasizing a view of health care providers that the cost of NIPT creates disparities in access to the test [27]. A focus on cost is also congruent with the findings of other studies of women’s views [10, 28, 29], including our own study of women and couples in Lebanon and Quebec [19].

Although we identified common barriers in both contexts, we still noticed discrepancies between ways HCPs perceived and addressed similar challenges. For instance, while cost was perceived as the main barrier to NIPT’s clinical introduction, the way HCPs tackled it diverged. HCPs in Quebec raised the cost issue from a justice, discrimination, and resource allocation lens, while HCPs in Lebanon focused on NIPT coverage by insurance companies.

Because the Quebec healthcare system is publicly-funded and prenatal tests are covered if medically indicated [30], Quebec HCPs were concerned about the consequences that lack of NIPT coverage^1^ might create, including concerns about equity issues related to who can access NIPT. They feared that the financial barrier might allow access to NIPT only to those who are better off, which would discriminate against those who are not able to afford the test, thus raising issues related to justice. This would run counter to the values endorsed by the Quebec universal health care system which is based on the State providing medical and hospital services to the population in an equitable manner.

To address this issue, some HCPs suggested that resource allocation is an important obstruct that should be considered when deciding about NIPT coverage. They proposed that for the moment, NIPT should be offered and covered for those women with high-risk pregnancies. This finding resonates with those in other studies where HCPs, as well as pregnant women [10], argued that NIPT offer and coverage should be provided for women with high-risk pregnancies.

While Lebanese HCPs raised cost as the most important barrier to NIPT’s clinical and ethical implementation, they discussed this issue in terms of lack of coverage by insurance companies. This could be explained by the fact that the Lebanese healthcare system is a hybrid one [20] and people must pay privately for prenatal tests if they do not have any form of health insurance. This means that if implemented in the current context, where there is a lack of public coverage for prenatal tests, NIPT might reinforce the existing disparities and inequities insofar as only those who are better off or who do benefit from a certain coverage will have access to the test.

In both settings, the lack of NIPT coverage creates access and equity issues, raising a challenge to its ethical implementation. HCPs in Quebec expressed concerns about these disparities more pronouncedly, since justice and equity of access are cornerstones of its universal healthcare system. In Lebanon, the absence of a public healthcare makes the impact of lack of coverage for NIPT less significant, when compared to the Quebec setting. Overall, our study demonstrated the need to address these concerns to ensure an equitable access to the test.

Another discrepancy was observed regarding pregnancy termination as a possible consequence of NIPT. In both settings, HCPs were concerned about possible increase in pregnancy terminations, however the reasons they invoked for such an increase were different. In Quebec, HCPs mentioned the increase in pregnancy terminations of affected pregnancies, to eliminate certain genetic particularities. They stated that this might lead to a decrease in the number of people with disability and a reduction in acceptance of them and their families. These issues have been well documented as concerns raised by the public, women, and their partners and scholars in many countries such as United Kingdom (UK) and the Netherlands [31–33]. These concerns might be explained by the fact that in Quebec, the existence of a universal prenatal screening program and the potential implementation of NIPT in this program, is/might be perceived as a ‘eugenic policy’ and a measure to eliminate or even ‘eradicate’ people with specific genetic conditions [34].

As for Lebanese clinicians, they were more likely to be concerned with an increase in pregnancy terminations related to sex selection for family balancing purposes, but not for affected pregnancies. This issue can be contextualized by the important weight placed on sex and gender, rather than on other diseases. This can be explained as follows: religious beliefs play a crucial role in patients declining the termination of an affected pregnancy, as mentioned by Lebanese HCPs. This finding confirms the results of a previous study, where the majority of women mentioned their religious convictions as a primary reason for declining testing and pregnancy termination [19]. However, in Lebanese society, and in some specific regions, the cultural value placed on gender, male favoritism, as well as family balancing, is of utmost importance. These preferences are reflected in clinical practice by the presence of clinics who offer IVF (in-vitro fertilization) for family balancing as one of the services they provide.^2^

The importance of sex and gender in the Lebanese context is further demonstrated by Lebanese HCPs’ acceptance of terminating pregnancies related to sex chromosome abnormalities. For instance, a study performed in 2002 by Zahed et al. showed that over 70% of Lebanese HCPs surveyed are in favor of terminating a pregnancy in the case of sex chromosome abnormalities such as Turner’s and Klinefelter’s syndromes. The authors classified these syndromes as relatively “severe” disorders, rationalizing this by stating that Lebanese society is one where “cultural importance [is] placed on sexual identity, fertility, and reproduction [35].”

The comparison of our findings in Lebanon and in Quebec points out the reasons underlying a similar concern raised by the routinization of NIPT: the potential increase in pregnancy terminations, which has been as well reported in other studies [36, 37]. Since prenatal testing is inevitably linked to the abortion debate - as it may lead couples and pregnant women to consider whether to continue or terminate a pregnancy, abortion policy in place is of utmost relevance.

Quebec and Lebanon have different policies shaping the availability of abortion. In Quebec, termination of pregnancy is not legally restricted at any point during pregnancy, although late term abortions (after 24 weeks of gestation) are rarely performed [21]. In Lebanon, abortion is illegal except in cases where the mother’s life is in danger, so it is not available based on NIPT results [22]. In Lebanon this means that if NIPT is offered routinely, it might result in an increase in clandestine and unsafe abortions.

A discussion of the ethical issues related to various reasons for pregnancy termination (e.g. medical versus non-medical) goes beyond the scope of this paper. However, the way NIPT will be implemented - whether in Lebanon or Quebec - should be approached carefully by counseling pregnant women and their partners about the test, and the options that are available to them following testing, to promote informed decision-making. As our findings show, HCPs raised continuing education and training of HCPs as a concern, and therefore it seems that it is an important factor to be taken into account in supporting women and couples in their decision-making.

On another note, the marketing of and the potential surge in NIPT prescriptions due to the incentives offered to physicians, was a concern raised by a Lebanese HCP. This concern reflects the dynamic between pharmaceutical representatives and physicians, characterized by an overall “culture of acceptance of gift-giving” (such as travel expenses to attend conferences, sponsoring of research) by physicians, which one participant described as influencing prescription practices [38]. This situation might have been exacerbated by the lengthy absence^3^ of a law, as well as a Lebanese code of ethics, to regulate the interactions between pharmaceutical representatives and physicians. In this context, ethical concerns arise regarding physicians being inclined to increase the uptake of NIPT to recoup financial gains. The presence of financial incentives related to the uptake of NIPT is thus a possible barrier to the implementation of ethical practices of informed decision-making.

Quebec clinicians did not raise industry involvement as a concern for NIPT implementation, which might be explained by the fact that physicians-industry’s representatives’ interactions are governed by strict rules that guide these relationships, along with other aspects such as prescriptions and research [39]. Therefore, NIPT will be regulated under the prenatal screening program and prescribed under specific conditions (for instance for women with high-risk pregnancies). Nevertheless, the literature shows concerns regarding NIPT development and dissemination through industry, leading to the test being marketed directly to clinicians, bypassing evidence-based and validation processes required for securing the quality assurance of the test [7, 10, 40].

Such context may lead to confusion on the part of HCPs who might lack the appropriate training and education about the use of the test. This, in turn, may negatively affect the counseling process provided to pregnant women or couples, and thereby their decision-making. This might be especially relevant in the Lebanese context, where the pressures and incentives to market NIPT might be more visible and pronounced. This issue could be addressed on two different levels. At the policy level, through the regulation of multinational biotech companies, which may be a challenging task given the competitive pressure to market NIPT. It may also be addressed at a micro-level, through the interaction and communication about prenatal testing that takes place between HCP and pregnant women or couples. This micro-level interaction is amenable to the training and education of HCPs, to ensure they are well prepared to help women and couples navigate the ethical complexities of NIPT.

The value of the cross-cultural comparison has been explained in detail elsewhere [19]. This comparison shows the importance of context, including the social, cultural and policy structures in place, in understanding the pressures that should be taken into account for an ethical implementation of NIPT. Our study attempts not only to discern some of the factors attributable to the differences and specific challenges related to each context, but also to the factors behind the common challenges. For instance, although HCPs voiced the same concerns regarding pregnancy terminations, their rationales were different, reflecting the way context, norms and values shape the understanding of the issues surrounding the implementation of NIPT.

### Strengths and limitations

A strength of this study is the combined qualitative and comparative approach, which allow for comparing and exploring in depth the views and perceptions of HCPs. However, we acknowledge that this study has limitations. Although the professional groups represented in the sample are diverse, there is a lack of medical geneticists and genetic counselors from Lebanon. This is due to the scarcity of medical geneticists in Lebanon and to the complete absence of genetic counselors. This difference in the composition of the samples might have affected the findings from the Lebanese context. Another limitation is that participants were recruited from specific units whether across Quebec or Lebanon. In Quebec participants were recruited from the obstetrics-gynaecology department and medical genetics division and in Lebanon from the maternal fetal division. The importance of the context in which HCPs practise might influence their views and perceptions and may therefore restrain the transferability of some of the findings to other settings or units.

## Conclusion

This study sheds light on the challenges for the clinical implementation of NIPT in Quebec and Lebanon. It highlights the existence of similar and different challenges in both settings, that are in turn influenced and shaped by the social, cultural and policy contexts in place. At a policy level, this reflects the need to consider cultural contexts in the development of guidelines to promote an ethically sound implementation of NIPT. Further, it shows that HCPs’ education and training remains paramount in order to provide NIPT counseling that supports pregnant women and couples’ informed choice.

## Supplementary information

**Additional file 1.** Interview guide for healthcare professionals in Lebanon and in Quebec. Interview guide used to collect data from healthcare professionals in Lebanon and in Quebec.

## Data Availability

The qualitative interview data analyzed during the current study are not publicly available because they might potentially include identifying information that could compromise research participant privacy and consent. Sections of anonymized data are available from the corresponding author on reasonable request.

## Abbreviations

AUBMC: American University of Beirut Medical Center
CBC: Complete blood count
CHUSJ: Centre hospitalier universitaire Sainte-Justine
CVS: Chorionic villus sampling
HCPs: Healthcare professionals
HP: Health professional
IVF: In-vitro fertilisation
Lb: Lebanese
NIPT: Noninvasive prenatal testing
Qc: Quebecois
UK: United Kingdom

## Footnotes

1 At the moment of data collection NIPT was still not covered by the Quebec healthcare system. However, in 2020 NIPT will start to be covered for high-risk pregnancies.

2 http://fakihivf-lb.com/treatment/family-balancing/

3 The “Code of ethics for medicinal products promotion in Lebanon and implementation procedures” was implemented only in 2016.
