# Supplementary material for: Implementation challenges for an ethical introduction of noninvasive prenatal testing: a qualitative study of healthcare professionals’ views from Lebanon and Quebec
Source: BMC Med Ethics. 2020 Feb 10;21:15. doi: 10.1186/s12910-020-0455-x (PMC7011468; doi:10.1186/s12910-020-0455-x)
Supplement: Supplementary file 1 — Additional file 1. Interview guide for healthcare professionals in Lebanon and in Quebec. Interview guide used to collect data from healthcare professionals in Lebanon and in Quebec. [file 12910_2020_455_MOESM1_ESM.docx]

## Additional file 1: Interview guide for healthcare professionals in Lebanon and in Quebec

I would like to know about you, your background and your professional experience as a …?

1-Before today, have you ever heard of Noninvasive prenatal testing? If yes, how?

2- What did you think about the test when you’ve heard about it? And what do you think about NIPT when you compare it to current screening and diagnostic prenatal tests?

3- In general, what are the reasons for which:

- You currently offer NIPT (If the healthcare professional already offers the test)

- You might consider offering NIPT (In case the healthcare professional does not currently offer NIPT but might offer it in the future).

4- When offering NIPT to your patients, in your opinion what information should be discussed with your patients?

5- In your opinion, when should this information be discussed with your patients?

6- In your opinion, beyond the information provided by the healthcare professional, how else should patients be informed regarding NIPT?

7- How much time do you think is required for a pregnant woman to make an informed decision about NIPT?

8- In your opinion, who do you think should give NIPT results back to the patient (be it negative or positive results)? And why?

9- How should patients receive NIPT results? (In person, by phone, etc)

10- In your opinion, should Informed consent for NIPT be given verbally or in writing?

And in your opinion what are the information that should be included in the informed consent form?

11- Since NIPT offer a higher specificity when compared to maternal serum screening test, do you think that the way of communicating the information regarding the test would be different?

12- What are the reasons that might influence your decision to offer or not to offer NIPT?

13- In your opinion, should NIPT be offered for all pregnant women (independently from their risk? Please explain.

14- In your opinion, should NIPT be offered/ available directly to the consumer?

15- Currently NIPT costs around [depending on the setting in question: 800 USD in Lebanon, and 600 USD in Quebec] in certain private clinics:

- Do you think that this will have an impact on the woman’s decision-making to consider the test?

- Do you think that NIPT should be covered by the healthcare system? And why?

16- If NIPT were covered by the healthcare system, do you think it should be covered for and explain why please:

- Only high-risk women?

- Women with an advanced maternal age?

- To detect aneuploidies (e.g. Down syndrome, trisomy 13, trisomy 18, Turner syndrome, Klinefelter syndrome)

- Monogenic diseases like Cystic fibrosis

- Late-Onset diseases

- Sex determination for medical reasons? For non-medical reasons?

- Paternity testing

17- If NIPT were to be covered by the healthcare system, do you think that women would feel pressure to take the test?

18- Do you think that NIPT should become part of routine pregnancy care? Why?

19- If NIPT becomes a routine test offered during pregnancy, do you think that it will have an impact on society? Explain please.

20- In your opinion, what are the barriers that might face an appropriate clinical implementation of NIPT? And how?

21- In your opinion, how should healthcare professionals (obstetricians/ gynecologists, medical geneticists, nurses, genetic counselors) be informed about advances in NIPT technology?

22- In the future, technological advance might allow to sequence the entire genome of the fetus at a reasonable cost. Would you be in favor of offering NIPT in order to know the entire genetic sequence of your patient’s fetus? If yes/ no, what would be the reasons behind your choice?
